# UAV-derived building heights and floor counts for the University of Texas at Dallas (July 2025 dataset)

**DOI:** 10.1016/j.dib.2025.112263

**Published:** 2025-11-12

**Authors:** Shuang Tian, Fang Qiu

**Affiliations:** University of Texas at Dallas, Geospatial Information Sciences (GIS), 800 West Campbell Rd. MS GR31 Richardson, TX, 75080, USA

**Keywords:** UAV photogrammetry, Building height estimation, Digital Surface Model (DSM), Digital Terrain Model (DTM), Zonal statistics, Campus-scale mapping, Floor count estimation, Urban morphology

## Abstract

This Data Article presents a campus-scale dataset of building heights and estimated floor counts for the University of Texas at Dallas (UTD). Heights were derived from an unmanned aerial vehicle (UAV) photogrammetry workflow using a DJI Mavic 3 Multispectral (M3M) and ArcGIS Drone2Map (2024.2.0) to generate a Digital Surface Model (DSM), Digital Terrain Model (DTM), and true orthomosaic at 0.024 m ground sampling distance (GSD). Building footprints from OpenStreetMap (OSM) were refined and used with zonal statistics in ArcGIS Pro to summarize per-building height metrics (mean, median, min, max, std, pixel count). Estimated floor counts were computed using a representative floor height of 4.10 m. Validation against 38 buildings with ground-truth building height achieved height RMSE of 0.72 m (R² = 0.97) and floor-count RMSE of 0.52 floors with mean bias +0.04 floors (R² = 0.72). The dataset includes 373 continuous drone images, raster images (DSM/DTM/), building vectors with attributes, CSV tables for zonal statistics and validation, ArcGIS Pro models and scripts/metadata for reproducibility.

Specifications Table

**Table:** 

Subject	Earth & Environmental Sciences
Specific subject area	UAV photogrammetry; building height estimation; campus-scale urban data products; remote sensing data; geospatial data; high-resolution map.
Type of data	JPG (Raw UAV images); GeoTIFF (DSM, DTM, orthomosaic); GeoPackage/Shapefile (building footprints with attributes); CSV (zonal statistics, validation); ipynb(Notebook/Script with arcpy); ArcGIS Pro (.aprx)/Drone2Map(.d2mx) (ArcGIP Pro model and Drone2Map model)
Data collection	DJI Mavic 3 Multispectral (RGB used for photogrammetry), flight on 2025–07–01; ArcGIS pro and Drone2Map 2024.2.0
Data source location	University of Texas at Dallas, Richardson, Texas, USA
Data accessibility	Repository name: figshare Data identification number:https://doi.org/10.6084/m9.figshare.29880422 **Author ORCID ID:** 0009–0009–3638–2059 Direct URL to data: https://figshare.com/s/572b501492bcf35281e5 UTD Building Heights Detection with Drone Data repository includes files: • 01 ArcGIS Pro Model (folder) • 02 DJI_202,507,011,551 (folder) • 03 Research Area(folder) • 04 BuildingShapefiles(folder) • 05 Building attributesVerified38.csv • 06 Fig. 5. TrueOrthoMap (TIFF format) • 07 Fig. 6. DSM model (TIFF format) • 08 Fig. 7. DTM model (TIFF format) • 09 Fig. 8. HightsDistribution (TIFF format) • 10 UTDBuildingHeights.ipynb • 11 README.txt
Related research article	None

## 1 Value of the Data

• Provides 373 original, spatially continuous, high-resolution UAV images at centimeter-level (2.4 cm) resolution, capturing plants, buildings, trails, parking lots/structures, and vehicles—valuable for geospatial research (GIS), deep learning, and machine learning applications.
• Enables generation of a high-resolution RGB map for a 0.327 km^2^ section of the UTD campus, supporting GIS research, facilities management, and urban planning studies.
• Offers a unique UAV dataset for remote sensing research and teaching.
• Supplies validated building heights and floor counts for use in urban analysis, evacuation planning, energy modeling, and digital twin applications.
• Supports benchmarking of lightweight, cost-effective UAV workflows against LiDAR- or satellite-based approaches using a 38-building validation set.
• Includes reproducible *arcpy notebook (.ipynb format)* code for generating zonal statistics and accuracy validation, useful for research and teaching.
• Provides complete metadata suitable for remote sensing/GIS labs, machine learning training, change detection, and 3D city modeling.
• Facilitates cross-campus comparisons and temporal monitoring through repeat flights using a standardized method.

## 2 Background

Accurate building height information is essential for applications such as urban planning, emergency response, 3D city modeling, earthquake effect estimation and environmental analysis [1]. However, obtaining high-resolution vertical data is often challenging, especially in locations where traditional methods—such as airborne LiDAR or ground-based surveys—are financially prohibitive or logistically difficult [1].

Advances in unmanned aerial vehicle (UAV) technology and photogrammetric processing have created cost-effective, flexible alternatives for producing detailed 3D datasets at high spatial and temporal resolutions [1,2]. UAV photogrammetry supports the generation of Digital Surface Models (DSM) and Digital Terrain Models (DTM), which can be combined to derive building heights with sub-meter accuracy [3].

The dataset presented here was compiled using a UAV-based workflow over the research area of the University of Texas at Dallas (UTD) campus. Imagery collected with a DJI Mavic 3 Multispectral (M3M) and processed in ArcGIS Drone2Map was integrated with GIS-based analysis in ArcGIS Pro. Vector building footprints from OpenStreetMap (OSM) [5] were used in zonal statistical analysis to summarize per-building height metrics. A standardized per-floor height assumption was also applied to provide corresponding floor counts. This openly shared dataset is intended to support reproducible research, teaching, and the development of urban datasets in contexts where detailed building information is unavailable [4].

## 3 Data Description

The data in the folder: UTDBuildingData in the ***figure*** (platform) file name UTD Building Heights Detection with Drone Data, there are 11 files in the repository as follows Table 1:

**Table 1:** Files in the repository.

Item No	Items name	Content Description	Quantity	Foramt
01	ArcGIS Pro Model (folder)	UTDBuildingHeightDetection model (ArcGIS Pro) and Drone2Map processing model	1	ArcGIS Pro
		drone2map model	1	ArcGIS Pro Drome2map
02	DJI_202,507,011,551 (folder)	Raw UAV imagery DJI_20,250,701,160,038_0001_D to DJI_20,250,701,160,809_0373_D	373	JPG (Drone Images)
03	Research Area(folder)	Research area boundary shapefile set ((includes .cpg, .dbf, .prj, .sbn, .sbx, .shp, .shx))	8	Shapefile
04	BuildingShapefiles(folder)	BuildingShapefiles shapefile set (includes .cpg, .dbf, .prj, .sbn, .sbx, .shp, .shx)	8	Shapefile
05	Building attributesVerified38.csv	Verified building height and floor attributes	1	.csv
06	Fig.5. TrueOrthoMap	True orthomosaic generated from UAV imagery over the UTD campus	1	TIFF
07	Fig.6. DSM model	Digital Surface Model visualization	1	TIFF
08	Fig.7. DTM model	Digital Terrain Model visualization	1	TIFF
09	Fig.8. HightsDistribution	Building height distribution across the UTD research area	1	TIFF
10	UTDBuildingHeights	Jupyter Notebook containing the Python workflow for data analysis, visualization, and validation	1	.ipynb
11	README	Repository instructions and metadata summary	1	txt

### 3.1 Key files and contents (Table 1)

**01 ArcGIS Pro Model (folder):** This folder contents two models: ArcGIS Pro (version 3.4) model named “*UTDBuildingHeightDetection.aprx*” and Drone2Map model named “*UTDBuildingHeightDetection.d2mx*”. These two models need ArcGIS Pro license and ArcGIS Drone2Map (version 20,242_DJI_193,091) advance license. *UTDBuildingHeightDetection.d2mx* model converted 373 drone images to true ortho map, and generated DSM and DTM models for farther heights analysis in ArcGIS Pro. UTDBuildingHeightDetection.aprx model conducts heights analysis and zonal statistics. It includes vector and raster layers.

**Fig. 1:**
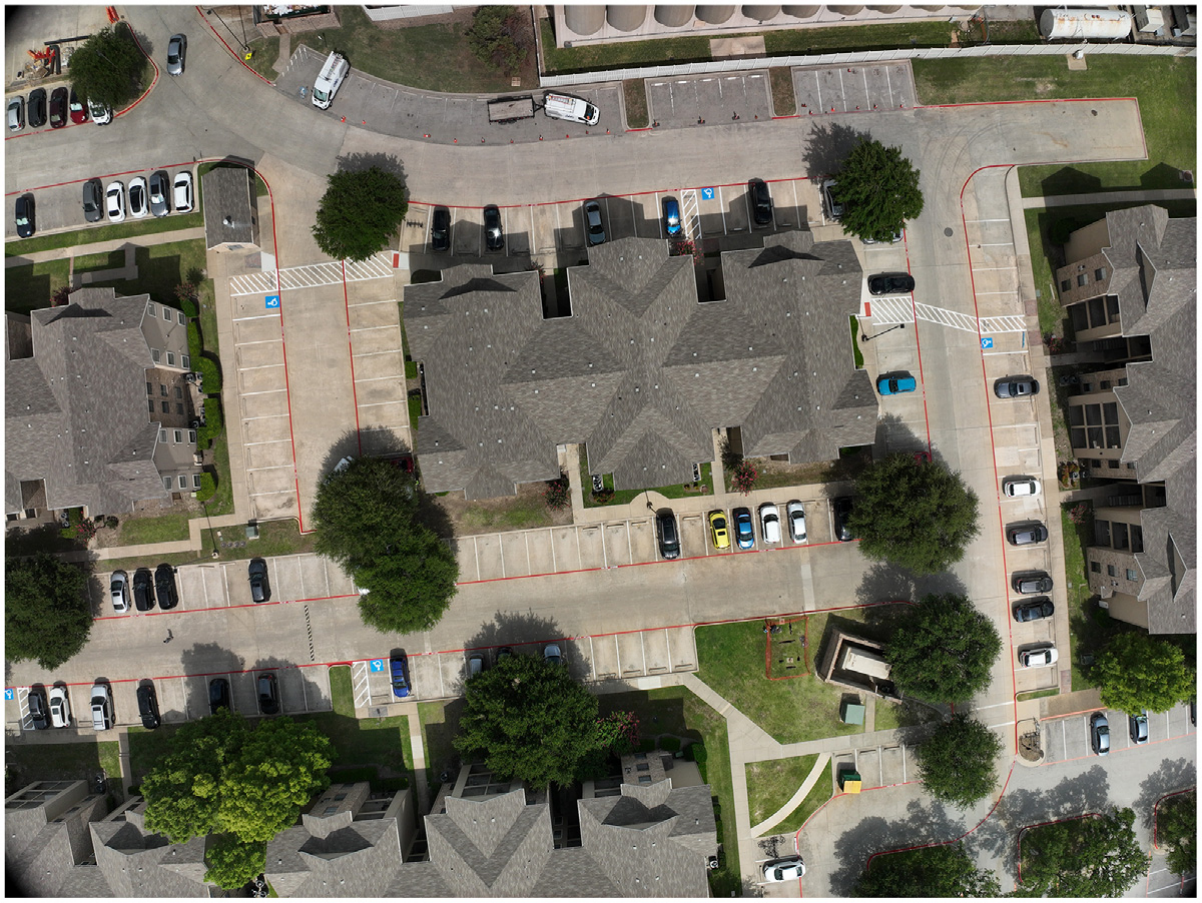
DJI_20,250,701,160,038_0001_image.

**02 DJI_202,507,011,551 (folder):** This folder contents high resolution 373 images (image number from *DJI_20,250,701,160,038_0001_D to DJI_20,250,701,160,809_0373_D*), which used to generate high resolution map, Fig.1. shows image-*DJI_20,250,701,160,038_0001_D*.

**03 Research Area (folder):** This folder contents building shape files (Note: a shape file includes “. cpg”, “.dbf”, “. prj”, “.sbn”, “.shx” “.shp” format files. In another word, if a shapefile is expected to be open, it must have these 6 format files simultaneously)

**04 BuildingShapefiles(folder):** This folder contents building shape files

(Note: a shape file includes “. cpg”, “.dbf”, “. prj”, “.sbn”, “.shx” “.shp” format files. In another word, if a shapefile is expected to be open, it must have these 6 format files simultaneously)

**Fig. 2:**
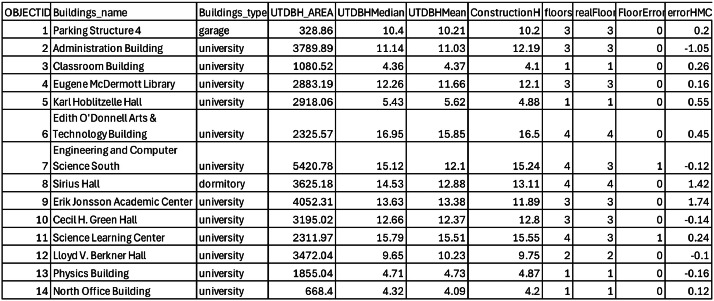
Part of Building attributesVerified38, which shows this file’s structure and attributes.

**05 Building attributesVerified38.csv:** This file includes columns (Fig. 2):

***OBJECTID*** *, unique Id, can be used to join data with building shape file attribute table.*

***Buildings_name*** *, buildings name, some have null values*

***Buildings_type*** *, such as garage, some have null values*

***UTDBH_AREA*** *, building footprint area, unit with meter.*

***UTDBHMedian,*** *derived a building’s height of median a raster height model, unit with meter.*

***UTDBHMean,*** *derived a building’s height of mean from a raster height model, unit with meter.*

***ConstructionH*** *, actual building heights, verified in field.*

***Floors, predits building floor per floor 4.10*** **m*.***

***realFloor, actual*** *building floor, verified in field.*

***FloorError,*** *FloorError = Floors- realFloor.*

***errorHMC,*** *errorHMC= UTDBHMedian- ConstructionH.*

This file contains verified height and floor count data for 38 buildings, used to assess the accuracy of building height and floor count estimations.

06 Fig. 5. **TrueOrthoMap** (TIFF format): This file generated from UAV imagery over the research area of UTD campus. The map provides a geometrically corrected, true-orthographic view of surface features and serves as a base reference for DSM and DTM products.

07 Fig. 6. **DSM model** (TIFF format): This file Digital Surface Model represents the elevation of object surfaces (e.g., buildings, vegetation) across the study area. Elevation values are expressed in **meters (m)**.

08 Fig. 7. **DTM model** (TIFF format)**:** This file Digital Terrain Model represents the ground surface elevations after removing buildings and vegetation. Elevation values are expressed in **meters (m)**

09 Fig. 8. **HightsDistribution** (TIFF format): This file **Height Distribution (PNG format):** Map showing the spatial distribution of building heights across the UTD research area, derived from the DSM–DTM difference. Height values are expressed in **meters (m)**.

10 **UTDBuildingHeights.ipynb** (Jupyter Notebook): This python notebook contains the full analytical workflow for data processing, statistical validation, and visualization of building height and floor count results. All code is executable in open-source Python environments (e.g., Jupyter Lab, Google Colab, Anaconda).

11 **README (txt format):** This document provides an overview of the dataset structure, file descriptions, coordinate reference system (WGS 1984 / UTM Zone 14 N), and usage instructions. It also outlines data sources, processing steps, and guidance for reproducing the analysis using the provided Jupyter Notebook.

### 3.2 Summary statistics

• Study area = 0.327 km^2^; 373 images processed.
• Validation set: 38 buildings; height RMSE 0.72 m, mean bias +0.04 m (R² = 0.97).
• Floor-count performance: RMSE 0.53 floors, mean bias +0.04 floors (R² = 0.73).

### 3.3 Data accessibility

Data and code are openly available in the Figshare repository at https://figshare.com/s/572b501492bcf35281e5

## 4 Experimental Design, Materials and Methods

### 4.1 Experimental design

**Fig. 3:**
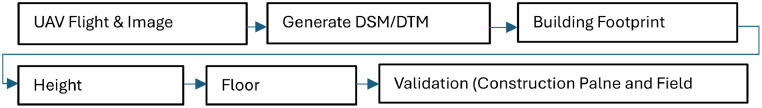
Schematic flowchart illustrating the complete UAV-derived building-height data-processing pipeline.

The experimental design of this study follows a systematic UAV-based workflow for deriving and validating building heights and floor counts across research area of the University of Texas at Dallas (UTD) campus. The overall process is illustrated in Fig.3 Schematic flowchart and consists of seven main stages:

**1. UAV Flight and Image Capture:**

A DJI Mavic 3 Multispectral (M3M) UAV was used to collect high-resolution RGB imagery over the study area (0.327 km^2^) under clear weather conditions. The flight plan was configured with **80** **% forward overlap** and **75** **% side overlap** to ensure adequate image redundancy for photogrammetric reconstruction.

**2. Generation of DSM and DTM Models:**

The captured images were processed in **ArcGIS Drone2Map (version 2024.2)** using Structure-from-Motion (SfM) photogrammetry to generate a **Digital Surface Model (DSM)** and **Digital Terrain Model (DTM)**. Both raster products were exported in **GeoTIFF format** with a spatial resolution of **0.024** **m** and georeferenced to **WGS 1984 / UTM Zone 14N**.

**3. Building Footprint Clipping:**

Building footprint polygons were extracted from **OpenStreetMap (OSM)** and refined manually to ensure completeness and spatial accuracy. Each footprint polygon was used to clip DSM and DTM values through **zonal statistics** in **ArcGIS Pro**.

**4. Height Calculation:**

Per-building height was calculated using the difference between the mean DSM and DTM elevations within each footprint:

(1) $$H_{building}=DSM_{mean}-DTM_{mean}$$

This step provided an estimated structural height for each building.

**5. Floor Estimation:**

The number of floors was estimated using a standardized per-floor height assumption of **4.1** **m/floor**, derived from local construction plans and building codes. Continuous and rounded floor estimates were both recorded.

**6. Validation (Construction Plans and Field Survey):**

Ground-truth building heights were collected from official **construction plans** and **field measurements** for **38 verified buildings**. These data were used to validate the UAV-derived results.

**7. Accuracy Assessment:**

Statistical evaluation was performed using the **Root Mean Square Error (RMSE)** and the **coefficient of determination (R²)** between UAV-derived and reference heights and floor counts. Validation results demonstrated strong agreement between the modeled and observed values.

### 4.2 Study area and acquisition

**Fig. 4:**
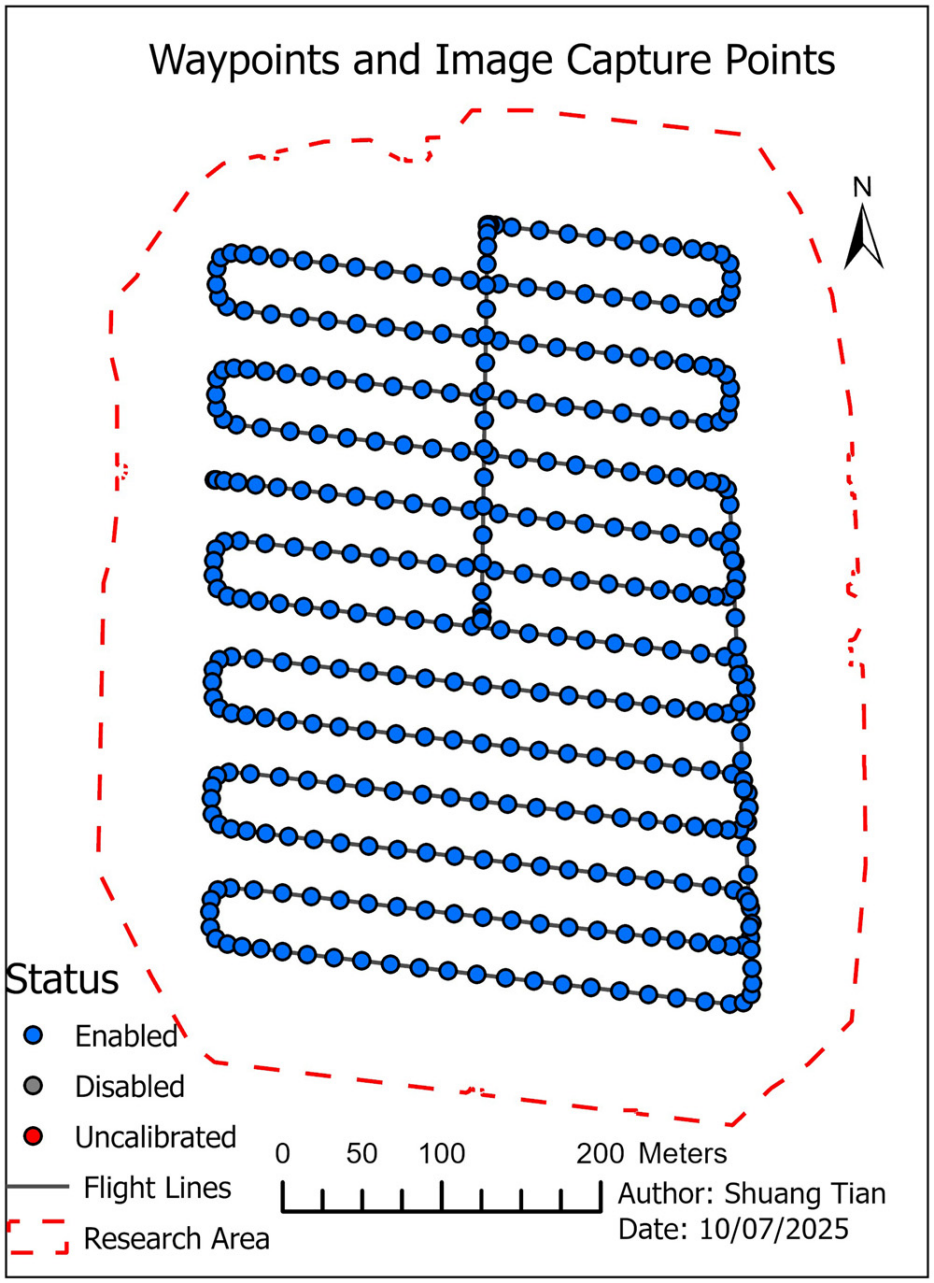
UAV flight plan showing waypoints and image capture locations (blue dots) distributed across the study area to ensure high image overlap for photogrammetric processing.

The primary dataset for this study was collected on 1 July 2025 using a DJI Mavic 3 Multispectral (M3M) unmanned aerial vehicle (UAV). The mission was piloted by the first author, **Shuang Tian**, who holds an FAA Certified Remote Pilot Certificate issued under Federal Aviation Administration (FAA) Part 107 regulations. The survey covered approximately 0.327 km^2^ of the University of Texas at Dallas (UTD) campus, encompassing 38 buildings, including Green Hall, Eugene McDermott Library, Administration Building, Naveen Jindal School of Management, Student Services Building, Parking Structures 3 and 4, and the Activity Canter, among others (Fig. 4) .

Flight operations were conducted between 16:30 and 16:55 CDT under cloudy conditions, with 5 statute miles visibility, an ambient temperature of 91°F, relative humidity of 55 %, wind speed of 4.63 m/s, and wind direction from 340° In compliance with FAA operational requirements, a trained visual observer, **Nicolas**, was present throughout the mission.

A total of 373 overlapping RGB images were captured (Fig. 4) and processed in ArcGIS Drone2Map (version 2024.2.0), achieving a ground sampling distance (GSD) of 0.024 m.

**Fig. 5:**
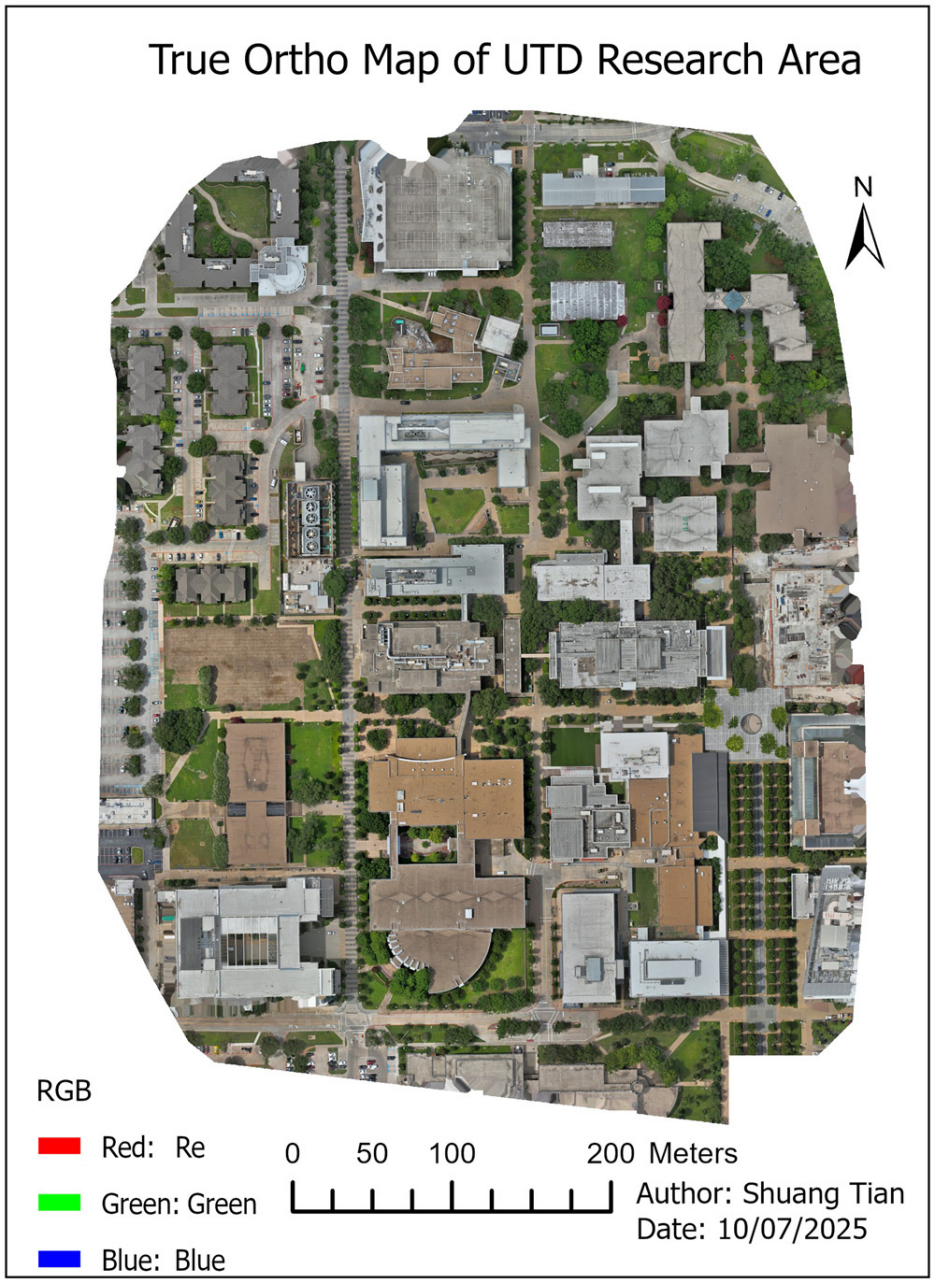
TrueOrthoMap: True orthomosaic generated from UAV imagery over the University of Texas at Dallas (UTD) campus. This orthomosaic serves as the base layer for DSM and DTM generation and allows precise spatial alignment of building footprints for height extraction.
• Digital Surface Model (DSM) (Fig. 6)

Processing in Drone2Map produced the following geospatial products (in the Drone2map model):

• True orthoimage (Fig. 5)

**Fig. 6:**
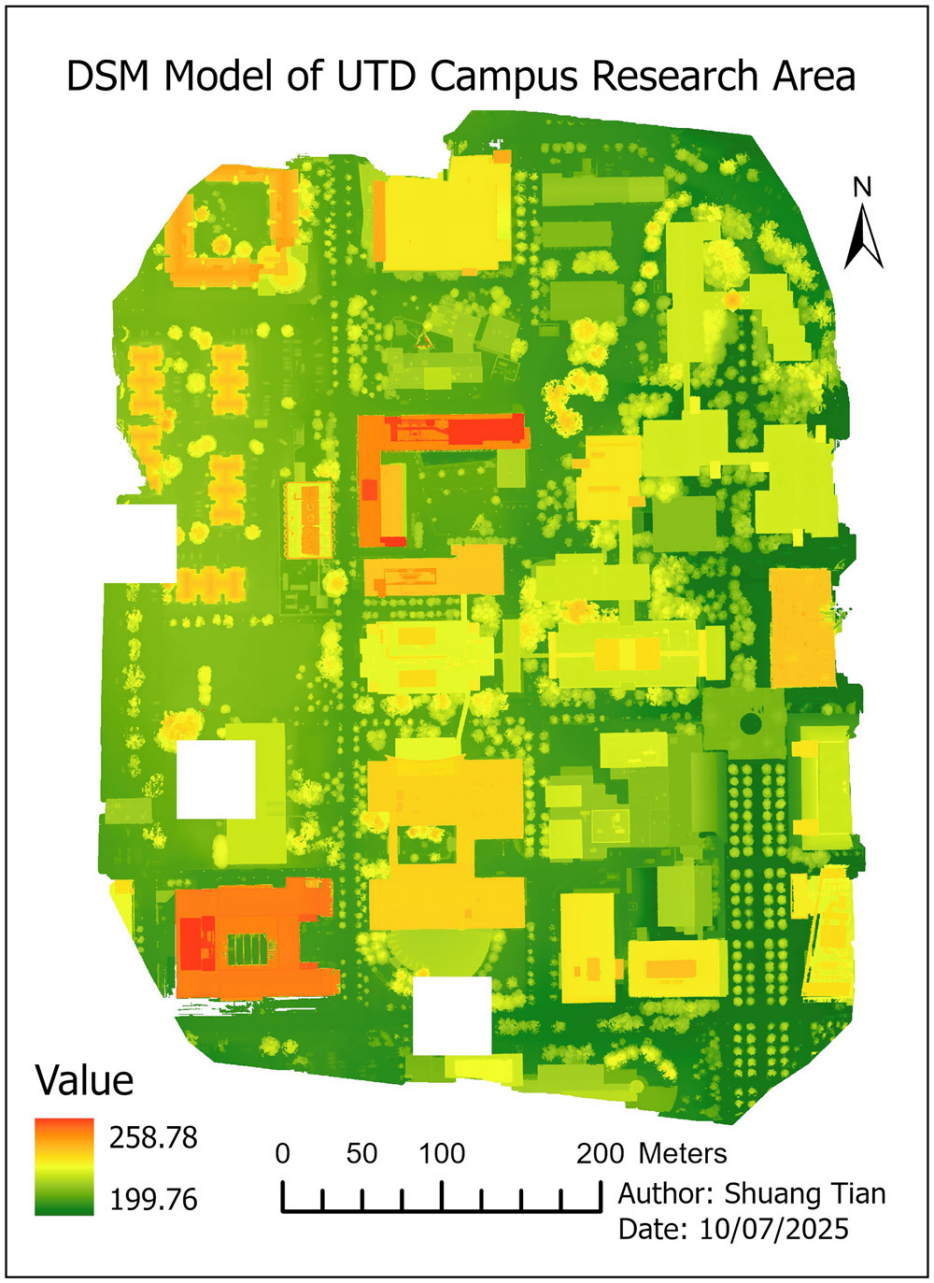
Digital Surface Model (DSM) of the study area, representing elevations of all visible surfaces, including buildings, vegetation, and other structures.
• Digital Terrain Model (DTM) (Fig. 7)

**Fig. 7:**
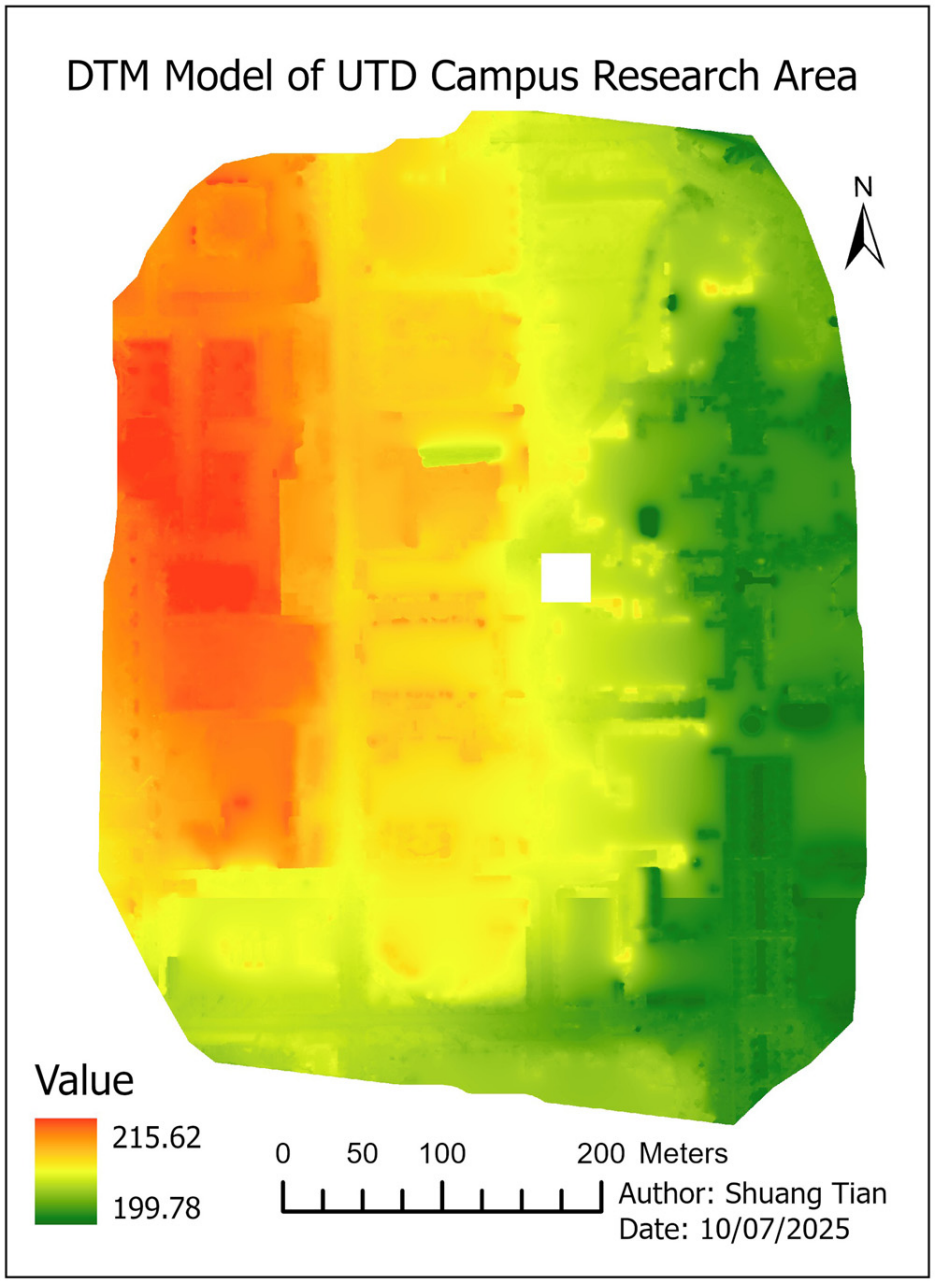
Digital Terrain Model (DTM) of the study area, representing the bare-earth elevation with buildings and vegetation removed.
• Height Distribution (Fig. 8)

**Fig. 8:**
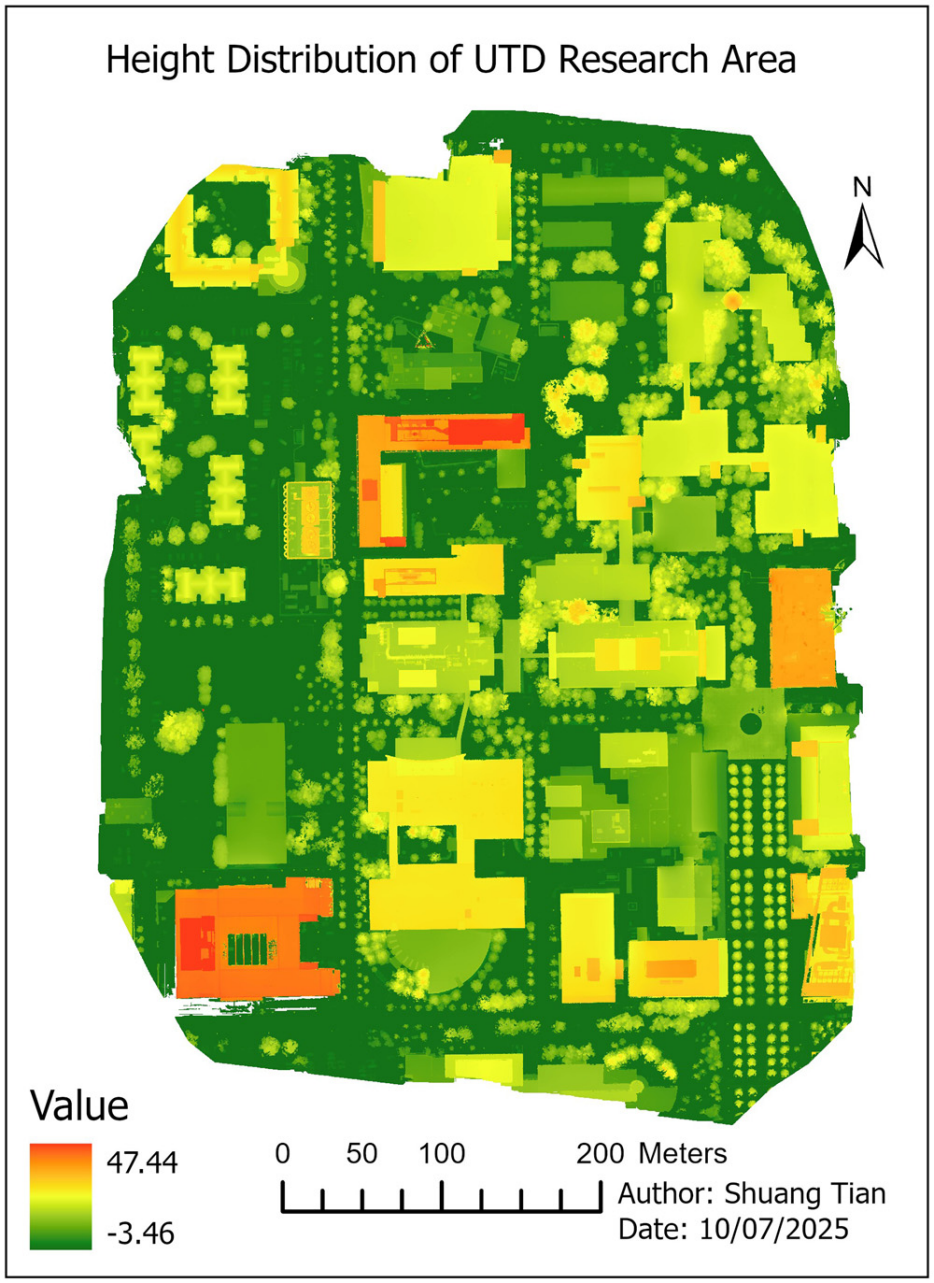
Building height distribution map derived from DSM–DTM subtraction. Red colours indicate taller structures; green cooler represents shorter buildings.

During image alignment, over 3 million tie points were identified, resulting in a reprojection root mean square error (RMSE) of 0.463 pixels, indicating high geometric accuracy. Processing times were approximately 11 min for DSM, 7 min for DTM, and 40 min for the true orthoimage.

For building-level analysis, OpenStreetMap (OSM) building footprints were retrieved and clipped to match the raster extent (Fig. 5), and the code detail please see the code in the notebook (.ipynb) the part “*1 clip the research area boundary*” (boundary in Fig. 4, red dash line was draw according to the Fig. 5’s raster layer boundary). These polygons served as analysis zones in ArcGIS Pro, where zonal statistics were applied to the height raster (DSM – DTM) to extract mean and median building heights. Estimated floor counts were then calculated by dividing the mean building height by the validated standard floor height of 4.10 m.

• Platform & sensor: DJI Mavic 3 Multispectral (RGB used for structure-from-motion).
• Date & time: 2025–07–01, UTC 20:50–21:10 (local: America/Chicago).
• Conditions: Cloudy; ∼33 °C; humidity ∼55 %; wind ∼4.6 m·s⁻¹ from 340°; visual observer present.
• Flight plan: Campus block near Drive H Road; overlaps and altitude configured for ∼0.024 m GSD (recorded in flight logs; details in README).

### 4.3 Photogrammetric processing

• Software: ArcGIS Drone2Map 2024.2.0.
• Outputs: True orthomosaic, DSM, DTM.
• CRS: WGS 84 / UTM Zone 14 N (EPSG:32,614).
• Horizontal and vertical units: meters; vertical reference per Drone2Map project.

### 4.4 Building footprints

• Source: OSM building polygons (download date recorded).
• Edits: snap/reshape to orthomosaic where necessary; remove non-building structures; ensure topology validity (no self-intersections).
• ID management: assign stable building_id for joining tables across releases.

### 4.5 Height model derivation

• Definition: *H* = DSM − DTM (meters) (equation 1).
• Raster math: performed in ArcGIS Pro Raster Calculator; nodata handling ensures only valid pixels within footprints are used.
• Smoothing (optional): light median filter to reduce rooftop noise (document parameter if applied).

The resulting building height distribution is illustrated in Fig. 8, showing the spatial variability of structure elevations across the UTD research area.

### 4.6 Zonal statistics

**Fig. 9:**
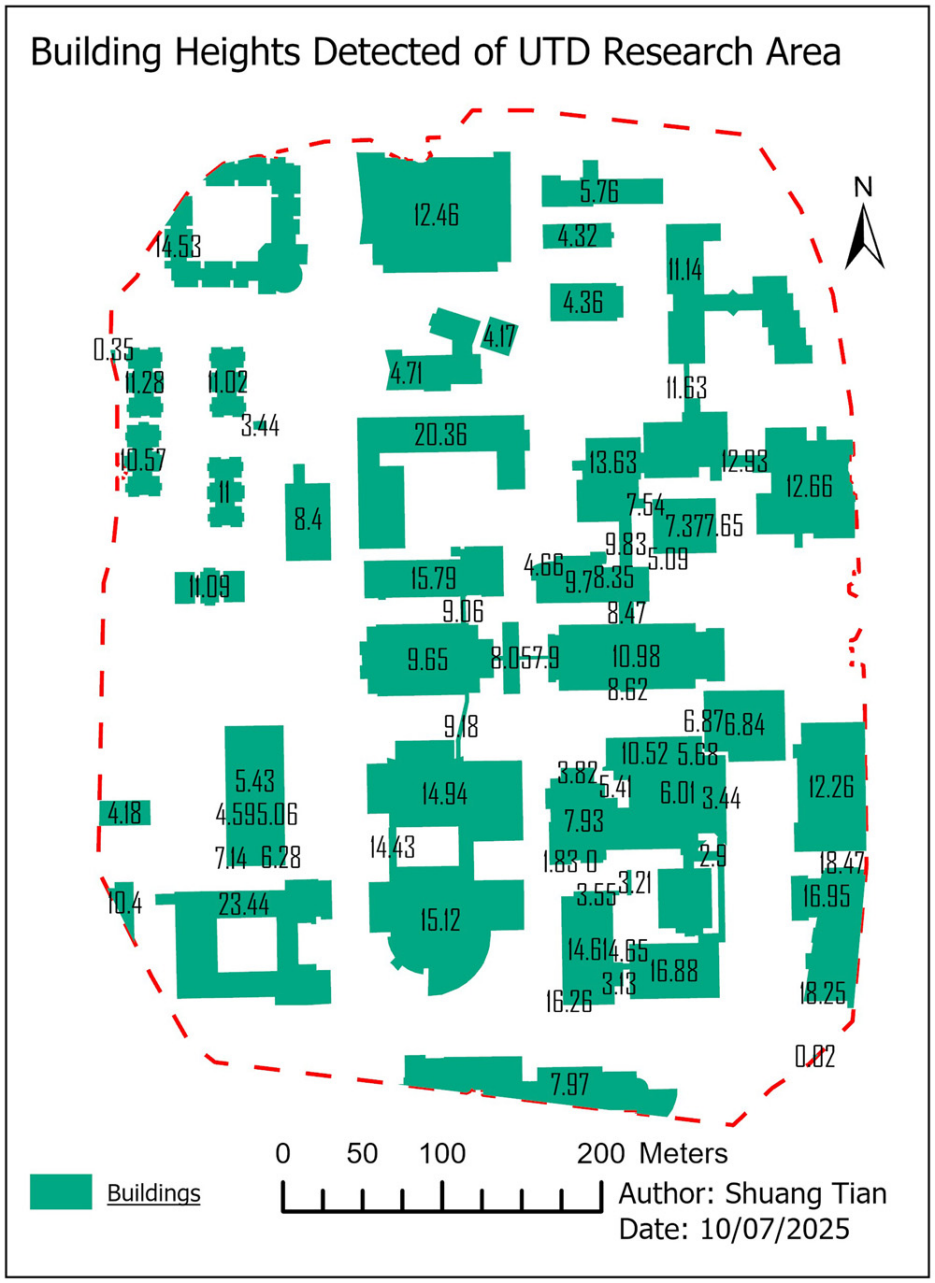
Extracted building-level height values using zonal statistics in ArcGIS Pro. Each polygon corresponds to an OSM building footprint, with median height values derived from the DSM–DTM raster layer.
• Output: GeoPackage layer and CSV export.
• Code details please see the code in the notebook (.ipynb) the part “2 Zonal statistics”.

• Tool: ArcGIS Pro Zonal Statistics as Table (statistics: mean, median, min, max, std; count).
• Mask: building footprints; input raster: H (DSM−DTM).
• Join: resulting table joined back to the buildings layer by building_id (Fig. 9).

### 4.7 Floor estimate

• Rule: est_floors_float = *h*_mean_m / 4.10.
• Integer floors: est_floors_int = round(est_floors_float) (nearest-integer rule).
• Sensitivity: Provide alternative columns for 3.6 m and 4.5 m per-floor assumptions if users need sensitivity analysis.

### 4.8 Validation

1. Ground truth: 38 buildings from facilities blueprints/records and field measurements.
2. Metrics: height vs. reference—RMSE, R²; floor counts—RMSE (floors), mean bias, R².
3. Plots: scatter (estimated vs. reference), residual histogram were generated; Accuracy assessment of metrics for height assessment are summarized in Table 2, while those for floor count estimation are provided in Table 3.

**Table 2:** Accuracy assessment of metrics (real height vs. predicted height).

Metric / Variable	Definition	Description	Value
errorHMC	UTDBHMedian − ConstructionH	Difference between UAV-derived and reference building heights	Vary
MSE	mean(errorHMC²)	Mean squared error (m²)	0.52
RMSE	$\sqrt{\text{MSE}}$	Root mean square error (m)	0.72
R²	1 − (SS_res_ / SS_tot_)	Coefficient of determination	0.97
Mean Error	mean(errorHMC)	Average bias (m)	0.01
n	-	Number of buildings validated	38

Note: **SS_res₎_** = ∑ (yᵢ − ŷᵢ) ², Residual Sum of Squares (error between observed and predicted values).

**SS_tot₎_** = ∑ (yᵢ − ȳ) ², Total Sum of Squares (variance of observed data).

**Table 3:** Accuracy assessment of metrics (real floor vs. predicted floor).

Metric / Variable	Definition	Description	Value
errorFloor	Floors − RealFloor	Difference between predicted and reference floor counts	Vary
MSE	mean(errorFloor²)	Mean squared error (floors²)	0.28
RMSE	$\sqrt{\text{MSE}}$	Root mean square error (floors)	0.53
R²	1 − (SS_res_ / SS_tot_)	Coefficient of determination	0.73
Mean Error	mean(errorFloor)	Average bias (floors)	0.04
n	-	Number of buildings validated	38

Note: **SS_res₎_** = ∑ (yᵢ − ŷᵢ) ², Residual Sum of Squares (error between observed and predicted values).

**Fig. 10:**
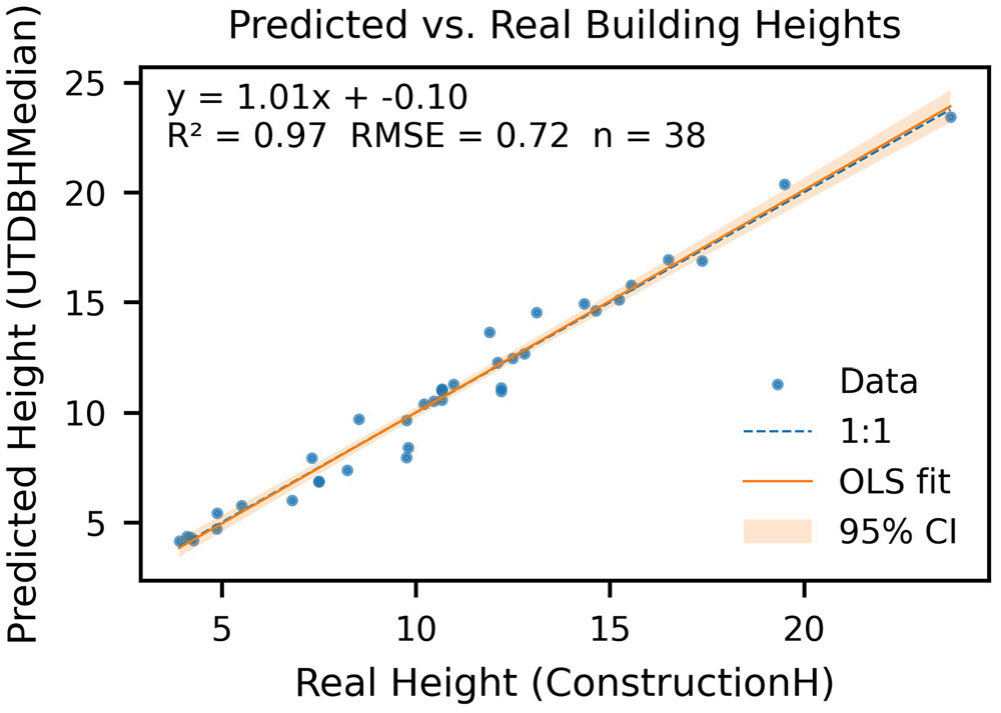
Scatterplot between field-measured and UAV-predicted building heights. The red line represents the ordinary least squares (OLS) regression fit, with the yellow shaded area indicating the 95 % confidence interval. The regression equation and R² are provided for reference.

**Fig. 11:**
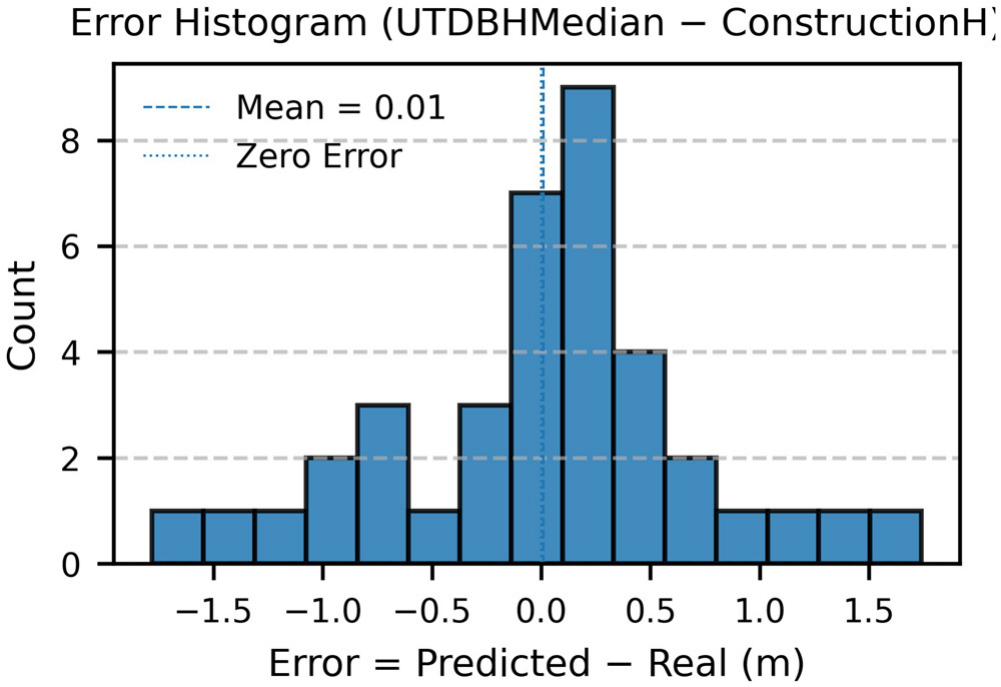
Error histogram (Predicted -real building Height). The means is near zero.

**Fig. 12:**
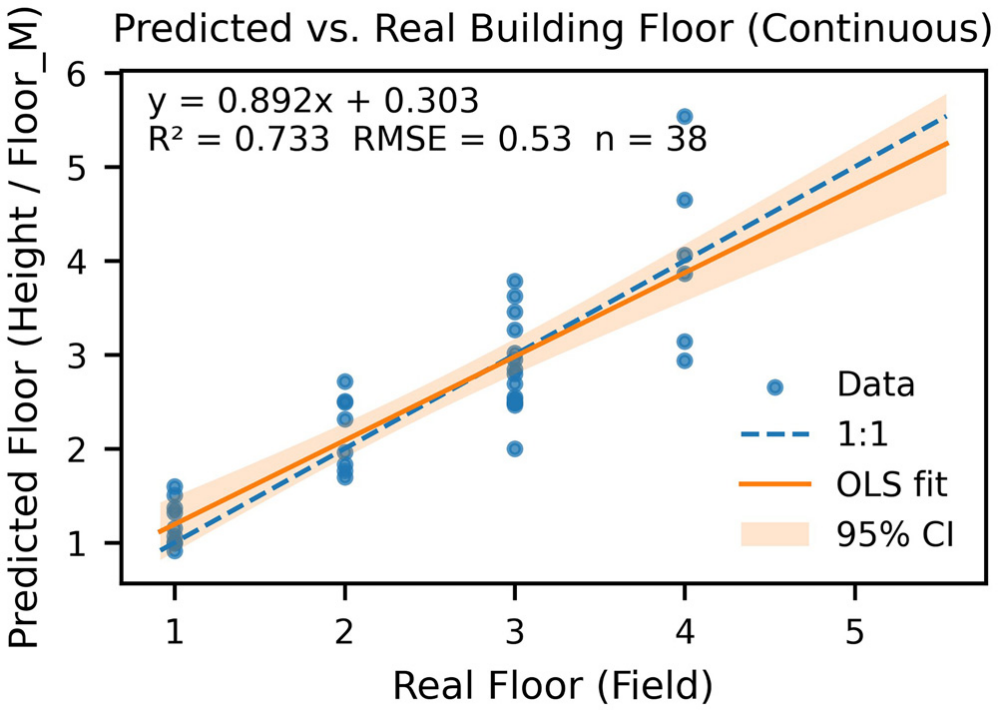
Scatterplot between field-measured and UAV-predicted building floors. The red line represents the ordinary least squares (OLS) regression fit, with the yellow shaded area indicating the 95 % confidence interval. The regression equation and R² are provided for reference.

**SS_tot₎_** = ∑ (yᵢ − ȳ) ², Total Sum of Squares (variance of observed data).
4. Findings: Building height achieved an RMSE of 0.72 m, a mean bias of +0.01 m, and R² = 0.97 (Fig.10, 11); Floor count achieved an RMSE of 0.53 floors, a mean bias of +0.04, and R² = 0.73 (Fig.12, 13).

**Fig. 13:**
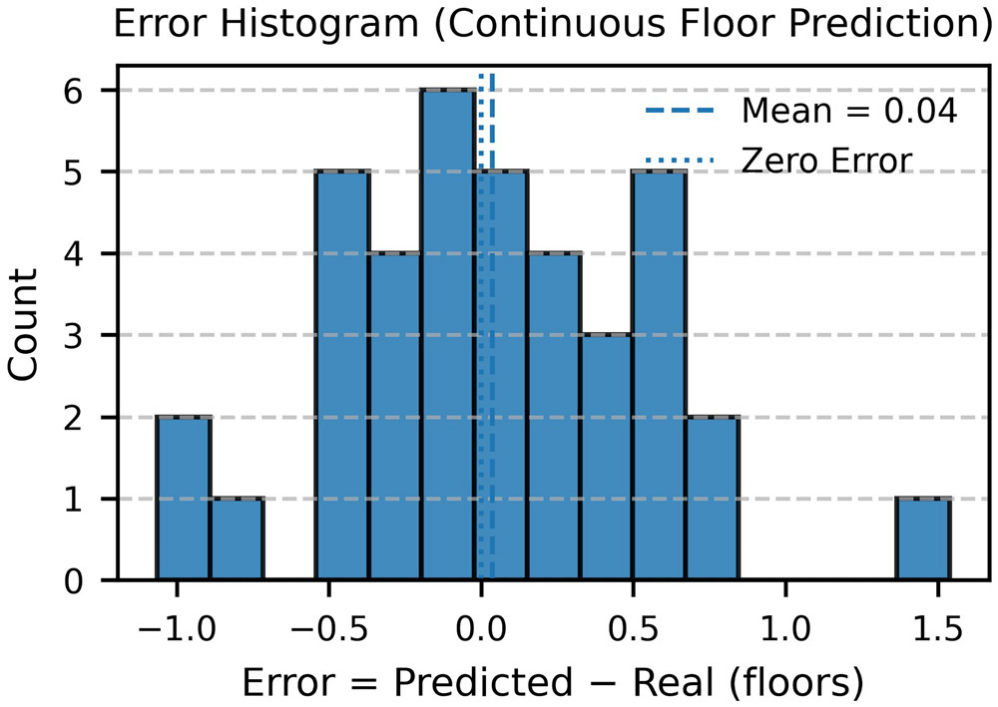
Error histogram (real floor vs. predicted floor). The means is near zero.
5. Code details please see the code in the notebook (.ipynb) the part “3 Validation for detection”

**Conclusion:** The validation results demonstrate that the UAV-derived building heights and floor counts are highly consistent with reference measurements, exhibiting minimal bias and strong correlation (R² > 0.7). These findings confirm the reliability of the proposed UAV–GIS-based workflow for accurate urban vertical information extraction.

**Conclusion:** The validation results demonstrate that the UAV-derived building heights and floor counts are highly consistent with reference measurements, exhibiting minimal bias and strong correlation (R² > 0.7). These findings confirm the reliability of the proposed UAV–GIS-based workflow for accurate urban vertical information extraction.

### 4.9 Reproducibility and open-source alternatives

The primary data processing and analysis were conducted using **ArcGIS Pro** (for spatial analysis and visualization) and **Drone2Map** (for photogrammetric DSM and DTM generation). To enhance reproducibility and accessibility, each component of the workflow can be replicated with open-source software. Specifically, UAV photogrammetric reconstruction in **Drone2Map** can be reproduced using **OpenDroneMap (ODM)**, which provides equivalent functionality for image alignment, point-cloud generation, and orthomosaic creation. Spatial analysis operations originally performed in **ArcGIS Pro**—such as raster calculation, zonal statistics, and attribute management—can be implemented in **QGIS** using corresponding tools (Raster Calculator, Zonal Statistics, Field Calculator). The accompanying **Python Jupyter Notebook (.ipynb)** is fully compatible with open-source Python environments (e.g., Anaconda, Google Colab, or Jupyter Lab) and does not require ArcGIS licensing. This structure ensures that all essential steps in the data-processing pipeline can be reproduced using freely available software.

## Limitations

**Known limitations:**

1. Complex rooflines and overhangs can lead to overestimation of building height measurements.
2. Vertical terrain variation on the campus causes building height and floor counts to vary even within a single building, making it difficult to determine exact values.
3. The campus spans both restricted airspace and Class G airspace (where drone flight is permitted), so UAV data collection was limited to areas within Class G.
4. OpenStreetMap (OSM) footprint completeness: the shapefile contains 75 vector polygons, but only 38 correspond to actual buildings, due in part to roof height variations and footprint discrepancies.
5. Differences in vertical reference systems compared to ground-truth measurements can introduce additional height errors.

To mitigate these limitations, several strategies are proposed for future improvement. Integrating **UAV-based or airborne LiDAR data** can enhance roof structure representation and height accuracy in complex architectural forms. **Harmonizing vertical reference systems** between UAV-derived models and ground-truth data will minimize systematic elevation differences. Additionally, **refining building footprints** through cross-validation with updated OSM layers and local cadastral or municipal GIS datasets can improve completeness and geometric precision. Collectively, these measures will enhance the dataset’s accuracy, consistency, and suitability for advanced applications such as urban modeling, 3D reconstruction, and change detection.

## Ethics Statement

The authors confirm that they have read and comply with the ethical requirements for publication in *Data in Brief*. This work does not involve human subjects, animal experiments, or any data collected from social media platforms.

During the preparation of this manuscript, the authors used ChatGPT (OpenAI) to assist in improving the grammar and fluency of the text. After using this tool, the authors carefully reviewed and edited the content as needed and take full responsibility for the integrity and accuracy of the final manuscript.

## Credit Author Statement

**Shuang Tian** was responsible for data curation, formal analysis, methodology, resources, visualization and writing original draft. **Dr. Fang Qiu** was responsible for conceptualization, investigation, project administration, supervision, validation, review and editing.

## Data Availability

figshareUTD Building Heights Detection with Drone Data (Original data).
